# Robust molecular subgrouping and reference-free aneuploidy detection in medulloblastoma using low-depth whole genome bisulfite sequencing

**DOI:** 10.1186/s40478-025-02049-1

**Published:** 2025-06-24

**Authors:** Dean Thompson, Jemma Castle, Martin Sill, Stefan M. Pfister, Simon Bailey, Debbie Hicks, Steven C. Clifford, Edward C. Schwalbe

**Affiliations:** 1 Wolfson Childhood Cancer Research Centre, Newcastle University Centre for Cancer, Newcastle Upon Tyne, UK; 2 Department of Applied Sciences, Faculty of Health and Life Sciences, Northumbria University, Newcastle Upon Tyne, UK; 3 Hopp Children´s Cancer Center (KiTZ), Heidelberg, Germany; 4 German Cancer Research Center (DKFZ) and German Cancer Consortium (DKTK), Heidelberg, Germany

**Keywords:** Medulloblastoma, Methylation, Microarray, WGBS, Sequencing, Aneuploidy, Low-pass, Subgrouping, Classification

## Abstract

Medulloblastoma comprises four principal molecular disease groups and their component subgroups, each with distinct molecular and clinical features. Group assignment is currently achieved diagnostically using Illumina DNA methylation microarray. Whole-genome sequencing (WGS) capacity is rapidly expanding in the clinical setting and the development of platform-independent, sequence-based assays of molecular group offers significant potential. Specifically, whole-genome bisulfite sequencing (WGBS) enables assessment of genome-wide methylation status at single-base resolution, however its routine application has been limited by high DNA input requirements, cost, and a lack of pipelines tailored to more rapidly-acquired and cost-effective low-depth (< 10x) sequencing data. We utilised WGBS data for 69 medulloblastomas, comprising 35 in-house low-depth (~ 10x) and 34 publicly available high-depth (~ 30x) samples, alongside cerebellar controls (*n* = 8), all with matched DNA methylation microarray data. We assessed quality (QC) and imputation approaches using low-pass WGBS data, assessed inter-platform correlation and identified molecular groups and subgroups by directly integrating matched/associated loci from WGBS sample data with the MNP classifier probeset. We further assessed and optimised reference-free aneuploidy detection using low-pass WGBS and assessed concordance with microarray-derived calls. We developed and optimised pipelines for processing, QC, and analysis of low-pass WGBS data, suitable for routine molecular subgrouping and reference-free aneuploidy assessment. We demonstrate that low-pass WGBS data can (i) be integrated into existing array-trained models with high assignment probabilities for both principal molecular groups (97% concordance) and molecular subgroups (94.2% concordance), and (ii) detect clinically relevant focal copy number changes, including *SNCAIP*, with greater sensitivity than microarray approaches. Low-pass WGBS performs equivalently to array-based methods at comparable cost. Finally, its ascertainment of the full methylome enables elucidation of additional biological complexity and inter-tumoural heterogeneity that has hitherto been inaccessible. These findings provide proof-of-concept for clinical adoption of low-pass WGBS, applied using standard WGS technology.

**Supplementary Information:**

The online version contains supplementary material available at 10.1186/s40478-025-02049-1.

## Introduction

Medulloblastoma (MB) is a high-grade embryonal brain tumour localised within the posterior fossa [1] and is one of the most common malignant CNS neoplasms of childhood [2]. Initial DNA methylation and expression profiling studies in MB [3–6] led to the identification of its four principal molecular groups (MB_WNT_, MB_SHH_, MB_Group 3_ and MB_Group 4_), each characterised by distinct molecular features and clinical behaviours [7]. These principal groups were incorporated into the WHO classification of CNS tumours in 2016 [8]. Since then, further studies using larger cohorts have revealed additional subgroups within MB_SHH_, MB_Group 3_ and MB_Group 4_ [9–12] which, in turn, were recognised in the WHO 2021 classification [1].

Assignment of MB molecular (sub) group using DNA methylation profiling was first established in 2013 [13] and is now a critical component of clinical diagnosis and research studies. A robust machine-learning brain tumour classifier, MNP (www.molecularneuropathology.org), has been widely adopted by the research community; as of 2024, > 100,000 brain tumour samples [14] have been assessed. This classifier relies on data arising from Illumina DNA methylation microarray technology, a cost-effective assay whose current release interrogates ~ 935,000 CpGs across the human genome and is compatible with both fresh-frozen and FFPE DNA derivatives [15]. Combining methylation array with targeted gene sequencing panels thus enables the identification of most clinically relevant molecular features associated with MB (i.e., copy number, mutation, molecular group).

Whilst methylation microarray technology is now firmly embedded in neuro-oncology diagnostics, there are limitations. For instance, specialist laboratory analytical infrastructure is required, and samples are run in batches; thus, costs and turnaround times may be impacted when smaller sample numbers are submitted – an issue for both rare tumour entities and smaller clinical centres [16]. Methylation array-based tools such as conumee [17] can also call copy number effectively, but the comparatively low resolution of even the most CpG-dense methylation array restricts reliable calling to regions where probe density is adequate and calling single copy/focal changes is unreliable. This hinders the detection of certain regions that are clinically relevant to MB, such as *SNCAIP* [10]. Focal gene amplifications critical in MB, such as *MYC* and *MYCN* are also under-called in comparison to gold-standard iFISH methods [18]. Importantly, any proprietary platform is dependent on its provider, presenting a risk of changes to the platform which may be incompatible with specific classifiers. This has happened three times since MNP was launched, as Illumina replaced the 450k microarray chip with EPIC, EPIC + and now EPICv2.0 versions. Each time this occurs, classifier retraining is needed, causing some disparity in tumour assignment with each new classifier version [19].

Whilst DNA methylation microarray is the current gold standard for brain tumour diagnostics, it is not the gold-standard means of assessing DNA methylation. Sequencing-based technologies such as whole genome bisulfite sequencing (WGBS) offer a means of interrogating the whole methylome (~ 28.3 million CpGs) at single-base resolution on a per-sample basis [20]. Despite being available since the 1990s, adoption of the technique has been limited due to prohibitively high DNA input requirements and economic costs. Recently however, new methodologies have been developed that render the platform a potentially attractive alternative to DNA methylation array in a research setting, with the costs of low-pass WGBS (< 10× coverage) becoming more comparable. Low-coverage methodologies are highly amenable to methylation sequencing, where the bivalent status of methylated and unmethylated reads enables the accurate calling of methylation status of specific CpG residues at lower read-depths. In theory, WGBS data outputs can be applied as direct substitutes to many in silico pipelines already developed for the array, including classification, offering a platform-independent means of group/subgroup assignment in MB and, through its genome-wide readouts, aneuploidy detection with increased coverage and sensitivity. However, this now needs to be tested in specific analytical contexts.

Genome sequencing infrastructure is increasingly being integrated within clinical diagnostic laboratories in high-income nations [21], presenting an opportunity for methylation-based sequencing to be used as part of routine brain tumour diagnostics. Indeed, methylation data derived from Nanopore sequencing has been utilised for classification of 46 brain tumour types [22], and reduced representation bisulfite sequencing (RRBS) has also been applied to cell-free DNA to derive tumour type with promising results [23]. However, both methods rely on model retraining and work with drastically reduced numbers of CpGs due to a lack of imputation. Another Nanopore-based study has utilised neural networks to deliver rapid diagnosis of subgroup within an hour of sequencing but has yet to reach high accuracy [24]. Given the rich resource (n > 100,000) of brain tumour methylation array samples upon which MNP is founded [14], and their potential for increased sensitivity and resolution, sequencing-based methods which can be integrated into existing array-based pipelines, are attractive.

In this study, we describe an optimised analytical pipeline for processing, quality control and analysis of MB WGBS data obtained from low-depth (< 10x) sequencing. We address missing data using imputation and demonstrate that molecular group/subgroup calls can be assigned with high probability using existing array-trained classification models. We further show that large-scale chromosomal alterations and focal regions can be identified with improved sensitivity to array-based methods using an optimised pipeline for reference-free copy number variation detection. Together, these provide important proof of concept for the adoption of platform-agnostic sequencing approaches into methylation-based diagnostics.

## Materials and methods

We performed WGBS on a representative cohort of 35 primary MBs at low pass (10 × cohort, DNA input = 2.5μg (*n* = 4), 150ng (*n* = 31). This was supplemented by 34 primary MBs and 8 normal cerebellar (4 adults, 4 foetal) samples sequenced at high depth (30 × cohort—ICGC), publicly available as pre-processed methylation beta values [25]. Methylation microarray data was available for all samples and was used for comparative analysis. Quality control and processing techniques for both data types are provided in the Supplementary Methods.

### Missing value imputation

To deal with missing data, both WGBS and DNA methylation microarray datasets were imputed. DNA methylation microarray data were imputed using the k-nearest-neighbours algorithm as previously described [25]. For WGBS data, we combined both datasets using bsseq v1.26.0 [26] and applied the XGBoost machine learning algorithm to impute continuous beta values between 0 and 1 via BoostMe [27]. Briefly, BoostMe prepares a training dataset for each sample, consisting of 1,000,000 randomly selected, non-missing CpGs above a user-defined depth (10× for this study), along with other parameters; distance to neighbouring CpG, nearest non-missing CpG methylation value upstream, nearest non-missing CpG methylation value downstream and average CpG methylation value of all other samples at the same loci, to ensure accurate prediction of missing data. Imputation model performance was assessed for each sample using the root mean squared error (RMSE).

### Investigating CpG variability

WGBS per-sample CpG methylation values that mapped to corresponding DNA methylation probe sites were correlated with their paired DNA methylation microarray sample using Pearson correlations. To investigate CpG variability and perform unsupervised clustering, we selected the 10,000 most variably methylated CpGs for each platform defined by standard deviation. These CpGs were then annotated to hg19 using the annotatr R package [28]. Any CpGs that shared the same annotation (*e.g.* located in the same CpG Island) were deemed non-unique. To determine overlap, the 10,000 most variable CpGs in our WGBS cohort were checked for overlapping ranges within the 450k, EPIC and EPICv2.0 probe manifests. As the WGBS platform surveys single CpGs and each probe assessed by the DNA methylation microarray spans ~ 50bp (Illumina), CpGs were defined as overlapping if they were within 25bp in both directions of a CpG site.

### Data visualisation and WGBS data classification

After variance filtering, both array and WGBS feature sets were visualised via principal component analysis (PCA) [29], uniform manifold approximation and projection (UMAP) [30] and t-distributed stochastic neighbour embedding (t-SNE) [31]. Cohorts were classified using MNP v12.8. For PCA, principal components were calculated for each feature set using the prcomp function within the stats v4.0.3 R package using default settings. UMAP was performed using the umap package v0.2.10.0 using a Euclidean distance metric and an n_neighbours value of 20. t-SNE was performed using the rtsne package v0.16 with a theta value of 0 over 100,000 iterations.

To investigate whether WGBS data was comparable with DNA methylation microarray data, we clustered our WGBS cohort alongside 426 publicly available medulloblastoma samples using molecular group calls from the original DFKZ MNP brain tumour classifier reference cohort (four principal groups of medulloblastoma, including a split of MB_SHH_ into infant and child/adult groups) [11]. We selected the same 32,000 features and group labels that were defined in the original reference set for clustering and clustered the data via t-SNE as previously described [14]. Finally, to test whether WGBS derived data was compatible with methylation array-defined classifiers, we applied our WGBS beta value matrix to the MNP classifier version 12.8 (https://epignostix.com). Aligned to current recommendations, a threshold of 0.7 for methylation sequencing data was used to define samples with confident classification [32].

### Chromosomal copy number analysis

Chromosomal copy number was estimated from WGBS-sequencing data using CNVKit v0.9.9 [33]. No specialised reference was used; the hg19 reference genome was supplied as an input in conjunction with the –no-edge parameter. To optimise calling, we increased bin size to 50,000bp and supplemented the current CNVKit filter regions list with additional regions including centromeric, telomeric and other known regions of poor mappability (Supplementary Methods). Copy number segments were derived, applying the –drop-low-coverage, –drop-outliers 100 and -t 1e-15 parameters. Following segmentation, we used MB_WNT_ samples with clear occurrences of monosomy 6 and MB_Group4_ samples with clear gain of chromosome 7 as a benchmark to empirically determine thresholds that define an absolute copy number integer where 0.3 defined an absolute gain, 0.7 an amplification, − 0.4 a loss and − 1.1 a deletion.

For matched array samples (*n* = 35), we performed aneuploidy detection using the conumee package as previously described [11]. 119 normal cerebellar samples were used as a reference for normalisation, obtained from the DFKZ paediatric brain tumour classifier reference cohort [14]. Platform-dependent concordance was assessed—segments were deemed concordant if greater than 50% of their length were overlapping. For 12 samples, copy number segmentation data from whole-exome sequencing was additionally available. For these samples, whole and single-arm copy number aberrations were identified using Nexus Copy Number 10.0 (BioDiscovery, El Segundo, CA, USA); a log2 ratio > 0.2 defined a gain and < − 0.2 a loss.

## Results

**Fig. 1:**
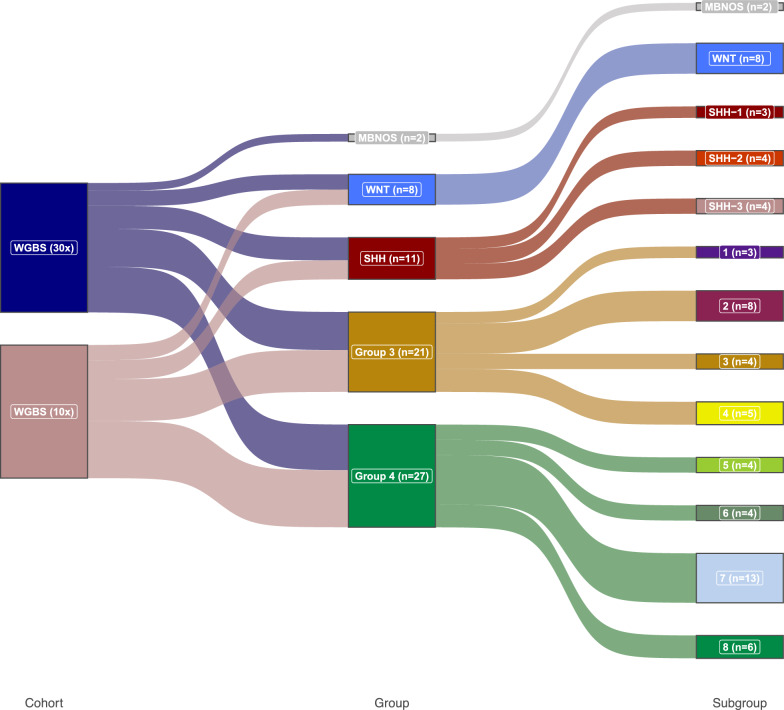
Molecular group and subgroup composition of study cohort. Sankey diagram of cohort with paired methylation microarray/WGBS data available, describing its molecular group and subgroup composition as determined by methylation microarray classification via MNP classifier version 12.8

Processing of matched methylation microarray and WGBS data (n = 69) yielded high-quality methylation data for downstream analysis (Supplementary Table 1). Beta value distribution differed between WGBS and microarray, owing to platform differences in how beta values are calculated (WGBS = read count-based calculation, Array = continuous intensity scale). As expected, both datasets were bi-modally distributed, with modal peaks close to 0 and 1 (Supplementary Figs. 1a–d). Molecular group, subgroup and other clinicopathological features are presented in Fig. 1 and Table 1.

**Table 1:** Clinico-pathological demographics of study cohort

Characteristic	ICGC cohort (%)	NMB cohort (%)	Combined (%)
**No. of samples**	34	35	69
*Sex*			
Male	**20** (58.8%)	**27** (77.1%)	**47** (68.1%)
Female	**14** (41.2%)	**9** (25.7%)	**23** (33.3%)
*Age at diagnosis*			
< 3	**4** (11.8%)	**3** (8.6%)	**7** (10.1%)
3–16	**29** (85.3%)	**30** (85.7%)	**59** (85.5%)
> 16	**1** (2.9%)	**2** (5.7%)	**3** (4.4%)
*Mean*	*7.66*	*6.86*	*8.93*
*Standard Deviation*	*4.65*	*3.57*	*11.28*
*Metastatic stage*			
M +	**20** (58.8%)	**23** (65.7%)	**43** (62.3%)
M-	**12** (35.3%)	**12** (34.2%)	**24** (34.8%)
Unknown	**2** (5.9%)	0	**2** (2.9%)
*Histology*			
Classic	**20** (58.8%)	**25** (71.4%)	**45** (65.2%)
Desmoplastic/Nodular	**5** (14.7%)	**3** (8.6%)	**8** (11.6%)
LCA	**9** (26.5%)	**7** (20%)	**16** (23.2%)
*Gene amplifications*			
*MYC* amplification	**1** (2.9%)	**4** (11.4%)	**5** (7.3%)
*MYCN* amplifcation	**2** (5.9%)	**3** (8.6%)	**5** (7.3%)
*Molecular group*^1^			
WNT	**4** (11.8%)	**4** (11.4%)	**8** (11.6%)
SHH	**6** (17.6%)	**5** (14.3%)	**11** (15.9%)
Group 3	**10** (29.4%)	**11** (31.4%)	**21** (30.4%)
Group 4	**12** (35.3%)	**15** (42.9%)	**27** (39.1%)
MBNOS	**2** (5.9%)	0	**2** (2.9%)
*Group 3/4 molecular subgroup*^1^			
Total No	22	26	48
1	**1** (4.6%)	**2** (7.7%)	**3** (6.3%)
2	**5** (22.7%)	**3** (11.5%)	**8** (16.7%)
3	**1** (4.6%)	**3** (11.5%)	**4** (8.3%)
4	**2** (9.1%)	**3** (11.5%)	**5** (10.4%)
5	0	**4** (15.4%)	**4** (8.3%)
6	**3** (13.6%)	**1** (3.8%)	**4** (8.3%)
7	**5** (22.7%)	**8** (30.8%)	**13** (27.1%)
8	**4** (18.2%)	**2** (7.7%)	**6** (12.5%)
MBNOS	**1** (4.6%)	0	**1** (2.1%)

^1^Molecular Group and Subgroup were determined using the MNP brain tumour classifier v12.8 (Capper et al., 2018)

**Fig. 2:**
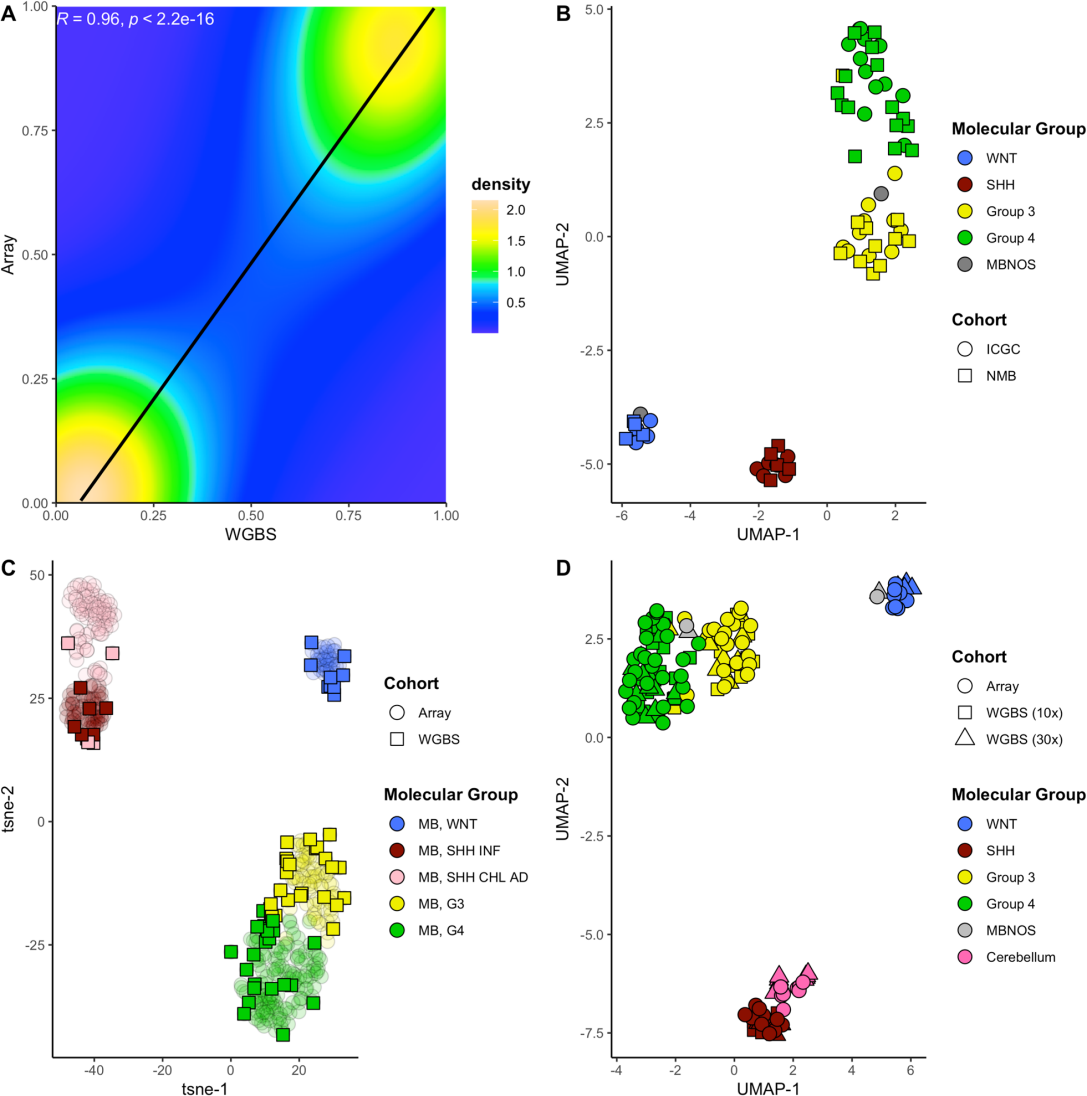
Methylation profiling of medulloblastoma using WGBS. **A** Density plot depicting Pearson correlation values between methylation microarray probe sites and corresponding WGBS CpGs (n = 422,027) between all paired samples in this study (n = 77 pairs). **B** UMAP plot of Array medulloblastoma profiles (n = 69) using the 10,000 most variably methylated loci. **C** t-distributed stochastic neighbour embedding (t-sne) plot of WGBS medulloblastoma samples projected on to the Capper et al., (2018) medulloblastoma reference cohort (n = 426) using CpGs that map to the original probe reference set (n = 32,000). **D** UMAP plot of combined WGBS and Array cohort (n = 69 medulloblastoma pairs, 8 normal cerebellum pairs). Sample shapes depict platform and colours denote molecular group

Mean cross-platform correlation between methylation microarray loci and matched WGBS CpG loci (*n* = 422,027) was high for both the 30X cohort (mean = 0.98, sd = 0.006), and the 10X cohort (mean = 0.94, sd = 0.0014; Fig. 2a, Supplementary Fig. 2a–b). The BoostMe imputation algorithm showed excellent performance, with RMSE between 0.09 and 0.22 (mean = 0.15). For 10 × data, an average of 4,576,687 CpGs were imputed (17.1%) and 282,858 for 30x (1.1%), Supplementary Table 2). Imputed values followed a continuous bimodal distribution, comparable to non-imputed test data (Supplementary Figs. 2c–d). After imputation, mean cross-platform correlation remained high for the 30X cohort (Mean cross platform Pearson’s correlation pre-imputation = 0.97, post-imputation = 0.96; Fig. 2a) and 10 × cohort respectively (pre-imputation = 0.94, post-imputation = 0.93).

As expected for the methylation microarray samples, the principal molecular groups of medulloblastoma were readily distinguished (Fig. 2b, Supplementary Fig. 3a–c). MB_WNT_ and MB_SHH_ tumours formed distinct clusters, whereas MB_Group3_/MB_Group4_ tumours clustered more closely together. Principal group assignments via MNP were congruent when clustering matched array and WGBS data together (Fig. 2c). Projecting imputed WGBS samples onto the MNP medulloblastoma reference cohort (*n* = 426) demonstrated that principal group assignments were maintained (Fig. 2d).

**Fig. 3:**
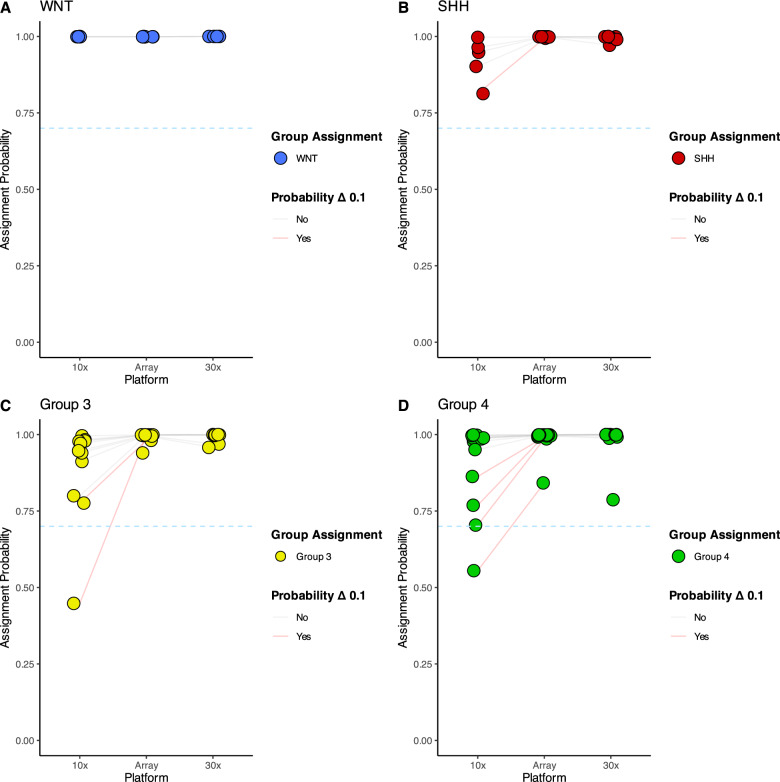
WGBS-derived methylation profiles are amenable to classification using MNP v12.8. Dot plots depict MNP v12.8 group assignment probabilities for matched methylation microarray/WGBS primary medulloblastoma samples at 10 × and 30 × coverage (*n* = 69) for **A** WNT tumours, **B** SHH tumours, **C** Group 3 tumours, **D** Group 4 tumours. Dots with lines spanning each platform indicate sample pair, with a blue dotted line spanning the plot denoting the 0.7 cutoff often used to confidently classify samples in research. Sample pairs that exhibited a classification probability change of 0.1 or greater are joined in red. Singleton samples without connecting lines did not have concordant classification calls

Applying WGBS medulloblastoma sample data to the MNP classifier (v12.8) gave high concordance with matched array data; 67/69 (97.1%) principal molecular group assignments were concordant across platforms, with 66/69 (95.6%) having a probability greater than 0.7 (Fig. 3a–d). Misclassifications included one 30X tumour classified as MBNOS, which was also MBNOS by methylation array (array probability = 0.238), and another 30X tumour which switched from a confidently classified MB_Group3-4_ by WGBS to a confidently classified MB_Group4-7_ by array (Supplementary Table 3 A). Principal group assignment probability was slightly lower for WGBS data compared to matched array samples (WGBS mean probability = 0.94, SD = 0.13/Array mean probability = 0.988, SD = 0.09, *p* < 0.001), and probabilities were significantly higher on average for 30 × data compared to 10x (30 × mean probability = 0.97; 10 × mean probability = 0.92, *p* = 0.042). Molecular subgroup calls were also highly concordant, with 63/69 (91.3%) with the same subgroup assignment, and 55/69 (79.7%) WGBS calls having a probability greater than 0.7 (Supplementary Table 3B). Assignment probability for WGBS data was lower than that of the array (WGBS mean probability = 0.85, SD = 0.19/Array mean probability = 0.96, SD = 0.13, *p* < 0.001). Overall, higher probabilities were observed for WGBS samples sequenced at 30 × compared to 10x (30 × mean probability = 0.90/10 × mean probability = 0.81, *p* = 0.027).

**Fig. 4:**
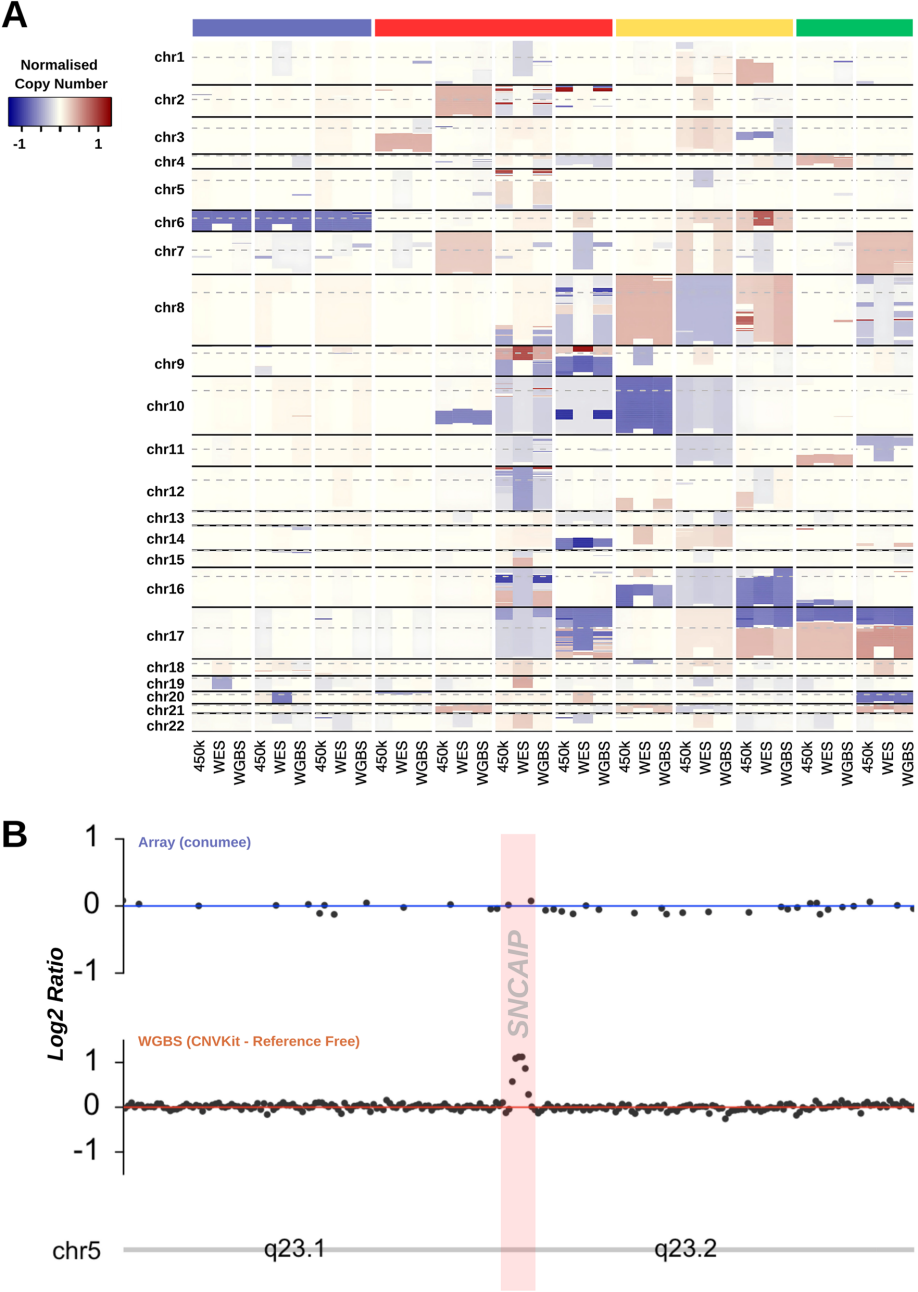
WGBS aneuploidy calls versus WES/450k derived estimates. **A** Heatmap depicting normalised log_2_ values derived from copy number analysis of WES, WGBS and methylation microarray data for 12 medulloblastomas with matched data available. Coloured bars indicate sample molecular group (WNT = blue, SHH = red, Group 3 = yellow, Group 4 = green). **B** *SNCAIP* duplication is detectable with WGBS but not with array-derived CN detection

CNVs detected from low-pass WGBS were typically concordant with matched methylation microarray samples. We observed a 96% sensitivity rate (where single arm/whole chromosome regions were called in both platforms), however 17/36 samples showed ≥ 1 mismatched segments comparing matched whole chromosome/single-arm copy number calls across the whole genome (Fig. 4a). Compared against matched WES-derived aneuploidy calls (*n* = 12), WGBS-derived calls achieved 94% sensitivity. Importantly, all copy number variation calls were highly comparable to that of the methylation microarray. For example, *MYC* amplification was reliably detected in two MB_Group3_ samples sequenced by WGBS, as well as *MYCN* amplification in two MB_SHH_ samples. In addition, focal regions of aneuploidy were identified using WGBS sample data that were not detectable from methylation microarray or WES data. A single *SNCAIP* duplication was also observed in a WGBS MB_Group4_, subgroup 6 tumour sample, which was not identifiable from analysis of its matched methylation microarray counterpart (Fig. 4b).

**Fig. 5:**
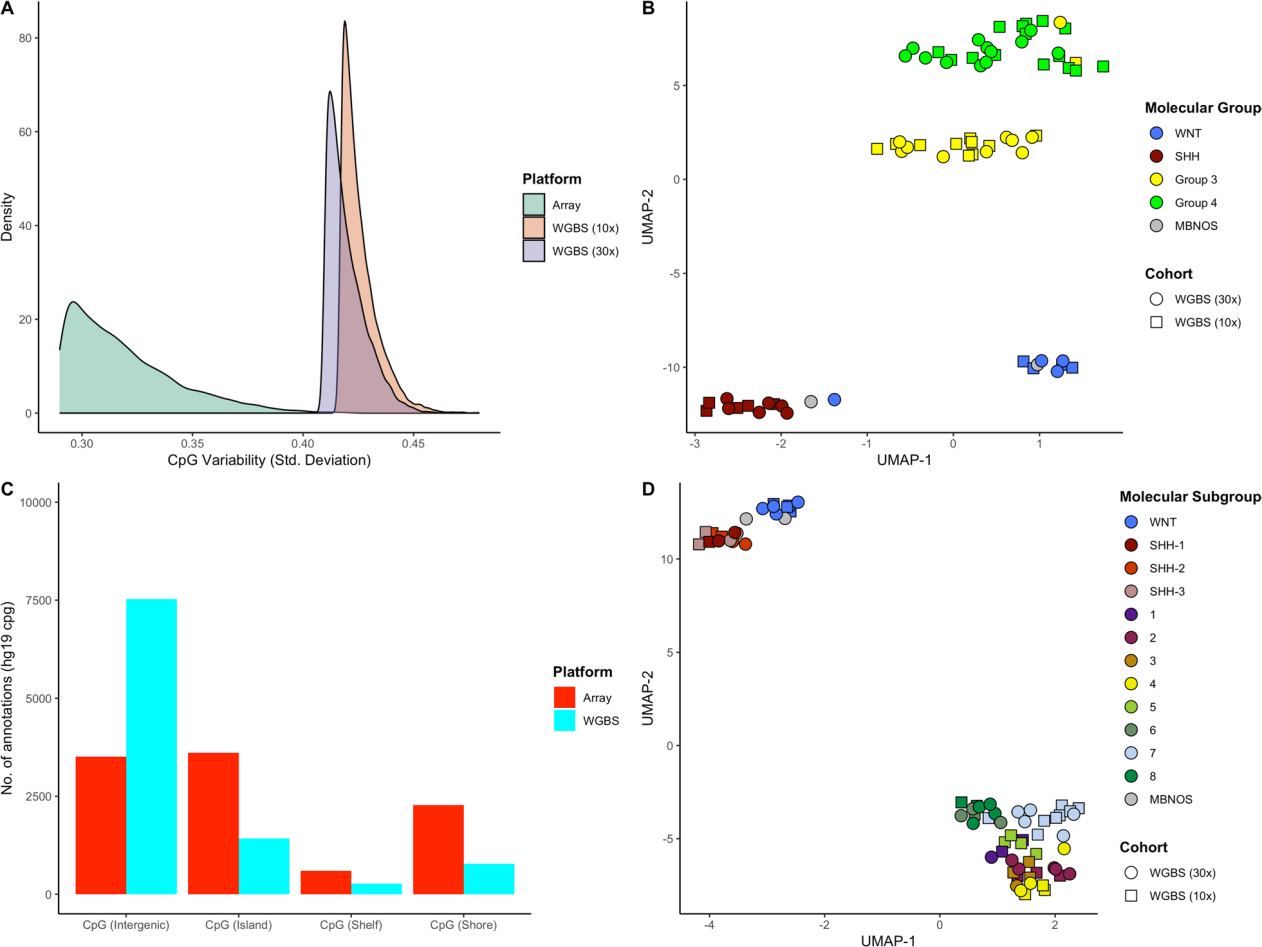
WGBS captures more variable CpG loci.** A** Density plot of 10,000 most variably methylated CpG/probe standard deviation values from methylation microarray (red), 10 × WGBS (orange) and 30 × WGBS (violet) samples (n = 69) **B** UMAP plot of WGBS medulloblastoma profiles (n = 69) using the 10,000 most variably methylated CpGs. **C** Barplot depicting the CpG annotation category distribution for the 10,000 most variably methylated CpGs/probes using array and WGBS methods, calculated using a standard deviation measure in the matched primary medulloblastoma cohort (n = 69). **D** UMAP plot of WGBS medulloblastoma profiles (n = 69) showing 2,644 most variable methylated CpGs that were mapped to unique genomic regions. Sample shapes depict cohort source and colours denote molecular subgroup

Characterisation of intergenic methylation and regions not covered by the methylation microarray revealed significant variability between the groups of medulloblastoma. The 10,000 most variably methylated CpGs in our WGBS cohort captured more variability than the 10,000 most variable CpGs in our DNA methylation microarray dataset, with sd values ranging between 0.40–0.46 and 0.17–0.43 respectively (Fig. 5a). The vast majority (9,502/10,000) of these variable CpGs in our WGBS cohort did not overlap with CpGs included in the EPICv2.0 probe manifest. Visualisation of the cohort using this feature set resulted in distinct separation of all four molecular groups of medulloblastoma, including groups 3 and 4 (Fig. 5b). Annotation of all 10,000 CpGs revealed that the vast majority of WGBS variable CpGs mapped to open sea regions (Fig. 5c), representing unexplored (sub)group-dependent methylation changes. Importantly, the most variably methylated CpG loci tended to lie proximal to other similarly variable CpGs; the 10,000 most variable CpG loci mapped to only 2,644 unique genomic regions (Supplementary Table 4, Supplementary Fig. 4a). Similarly, the 10,000 most variably methylated probes from methylation array mapped to 5,773 unique regions and the 32,000 CpG loci used as a classifier reference set in Capper et al., 14 mapped to 16,052 regions (Supplementary Table 5, Supplementary Fig. 4b). Restricting analysis to a single most variable CpG representative from each unique region and re-clustering resulted in a clustering more similar to transcriptome clustering of medulloblastoma, where groups 3 and 4 are less distinct and cluster along a continuum (Fig. 5d, Supplementary Fig. 4c) [34].

## Discussion

This application of low-input (150ng DNA), low-coverage WGBS for robust molecular (sub)grouping and CNV detection, provides important proof-of-principle for the platform-independent molecular subclassification of medulloblastoma tumours using DNA methylation data.

Methylation values obtained from samples sequenced < 10 × were highly correlated with data from matched methylation microarray samples, and imputation of missing data via machine learning algorithms was effective as part of model training. Ultimately, this yielded low-depth data from medulloblastoma tumours that was entirely compatible with pre-existing array-trained classifier models, circumventing the need for use of specific profiling technologies or platform-specific classification tools. Given the capability of the MNP classifier to predict the class of over 80 tumour entities, it is highly likely that data from other CNS tumour types sequenced using WGBS could also be used successfully for classification. This has the advantage of enabling accurate classification from sequencing data by leveraging the huge amounts of data already derived using methylation microarray. Indeed, given that methylation data derived from both platforms is analysed in analogous formats (Beta/M-values), methylation data from sequencing sources could straightforwardly be integrated into methylation array cohorts for analysis.

WGBS offered superior aneuploidy detection, since coverage is pan-genome and not at pre-determined probe sites of interest, which favour genic regions. The optimised reference-free CNVKit pipeline employed in this study was capable of reliably identifying focal regions of aneuploidy (including amplifications/deletions and single-copy gains) and called whole/single-arm regions with excellent sensitivity compared to matched methylation microarray and WES data, even with low-coverage data. Sequencing platforms such as WGS and WES can be used to identify focal amplifications across the genome but do not offer the capability to analyse methylation simultaneously.

Using WGBS, significantly higher numbers of variably methylated CpGs were identified that are not assayed by current methylation microarray platforms. Technical differences between platforms can partly help to explain this, where beta values derived from sequencing are strongly bimodal at 0 and 1, whereas array probe-based intensities typically range between 0.1 and 0.9. Coverage was important, with fewer reads naturally resulting in a smaller number of (un)methylated CpG counts for methylation beta value calculation. Despite these technical differences, the greater number of variable CpGs is likely due to full genome coverage, with many variable regions located in intergenic regions which lie outside the methylation array manifest. Clustering of these regions revealed distinct separation between the groups of medulloblastoma, including the transcriptionally similar MB_Group 3_ and MB_Group 4_. Further interrogation revealed that many of the most variable CpGs used for visualisation and molecular group definition in both array and WGBS datasets were proximal. This suggests that the absolute separation of MB_Group 3_ and MB_Group 4_ medulloblastoma previously associated with genome-wide DNA methylation, that is less evident from transcriptome studies [34, 35], may be a consequence of over-weighting certain genomic regions due to a multiplicity of highly variable, correlated probes that map to the same region.

Taken together, these findings demonstrate that the use of low-pass WGBS is directly comparable with array-based tumour subgrouping in medulloblastoma. WGBS, and other sequencing assays that employ nanopore sequencing or capture-based methods offer a platform-independent means of molecular subgroup identification that medical institutions could use with existing in-house sequencing infrastructure in developed nations. This would drastically decrease turnaround time to reporting, enabling clinicians to quickly make critical treatment decisions for patients. Moreover, the WGBS platform clearly offers superior copy number estimation [36].

The lower cost of using low-pass WGBS must be weighed against its limitations. Broadly, 10 × assignment probabilities were lower than 30 × WGBS, although high confidence assignments suitable for subgroup assignment were maintained. The lower read depth precluded accurate mutation and loss of heterozygosity inference that would be possible at higher sequence depth, however this limitation is also true for 30X data—the potential for unmethylated cytosines to confound C/T substitutions necessitates a higher read depth compared to conventional WGS. Likewise, accurate detection of mutations using WGBS, including those at low frequency is more problematic and panel-based methods that provide very-high (*e.g.* 1000-fold) coverage remain necessary.

As a technique, WGBS also needs consideration alongside emerging methylation assays using TWIST capture-based sequencing [37] and nanopore [38]. TWIST has the advantage of lower input amounts (as low as 20ng DNA) and is compatible with fragmented DNA from FFPE derivatives, but is limited by its incomplete genome coverage, reporting methylation at 3.98 million CpGs. CNV detection is possible, although detection of focal variants such as gain of *SNCAIP* is unclear. Given sufficient sequencing time, nanopore offers complete genome coverage and is also compatible with FFPE DNA derivatives, but has increased sample requirements (400ng relative to 150ng for WGBS), focal copy number changes were not detectable [37] and has lower accuracy than WGBS. While not assessed in our study, protocols for WGBS assessment of FFPE tissues are well established [39].

Despite continuing advances in methylation-based tumour classification, for example using Nanopore for intra-operative classification [24], whether there is an immediate appetite for paediatric oncologists and researchers to immediately transition from methylation array is unclear, given its low cost and the vast resources collated by MNP/Epignostix since its launch. Continuing technological advancements in sequencing, such as the planned 2026 launch of Illumina’s 5-base sequencing, enabling simultaneous CpG methylation and variant detection will address some WGBS limitations. However, its cost remains unclear and until methylation sequencing data resources become more comprehensive, it remains prudent to continue to take advantage of the large amounts of pre-existing array-derived sample data. Integration of WGBS subgroup classification into pre-existing array classifier models is straightforward and effective – such an integration is an important, necessary step to take advantage of platform-independent methylation sequencing technologies. The use of this assay, we believe, would ensure a smooth transition to such platforms in the coming years.

Herein, we describe an optimised, platform-independent analysis framework for processing, quality control and analysis of clinical medulloblastoma samples sequenced using WGBS at low depth. Our pipeline includes imputation, addressing the limitation of data missingness and, which when applied, provides high quality sample data suitable for both high-resolution aneuploidy detection and the accurate assignment of molecular group/subgroup using established, DNA methylation microarray derived classifier models, providing a proof of principle for its suitability for routine brain tumour diagnostics.

## Supplementary Information

Supplementary material 1

Supplementary material 2

Supplementary material 3

Supplementary material 4

Supplementary material 5

Supplementary material 6

Supplementary material 7

Supplementary material 8

## Data Availability

Data is available from the authors on reasonable request.

## Abbreviations

WGS: Whole genome sequencing
WGBS: Whole genome bisulfite sequencing
DNA: Deoxyribonucleic acid
QC: Quality control
MNP: Molecularneuropathology
CNS: Central nervous system
MB: Medulloblastoma
WHO: World Health Organisation
FFPE: Formalin-fixed parrafin-embedded
iFISH: Interphase fluorescent in-situ hybridisation
RRBS: Reduced representation bisulfite sequencing
ICGC: International Cancer Genome Consortium
RMSE: Root mean squared error
PCA: Principal component analysis
t-SNE: T-distributed stochastic neighbour embedding
UMAP: Uniform manifold approximation and projection
DFKZ: Deutches Krebsforschungzentrum
CNV: Copy number variation
