# Supplementary material for: Robust molecular subgrouping and reference-free aneuploidy detection in medulloblastoma using low-depth whole genome bisulfite sequencing
Source: Acta Neuropathol Commun. 2025 Jun 24;13:132. doi: 10.1186/s40478-025-02049-1 (PMC12186449; doi:10.1186/s40478-025-02049-1)
Supplement: Supplementary file 1 — Supplementary material 1 [file 40478_2025_2049_MOESM1_ESM.pdf]

<u>Supplementary Figure Legends</u>

Supplementary Figure 1:

Example density plot depicting the distribution of **A)** WGBS CpG methylation beta values for sample NMB_169, **B)** WGBS CpG methylation beta values for sample ICGC_MB140, **C)** Array methylation beta values for sample NMB_169 and **D)** Array methylation beta values for sample ICGC_MB140.

Supplementary Figure 2:

Density scatter plot depicting the inter-platform correlation of 10,000 randomly sampled beta values between all samples for all WGBS samples sequenced at **A)** 10x coverage and **B)** 30x coverage. **C)** Density plot depicting the beta value distribution of 10,000 randomly selected imputed (cyan) and actual (red) CpGs for all 10x sample data. **D)** Density plot depicting the beta value distribution of 10,000 randomly selected imputed (cyan) and actual (red) CpGs for all 30x sample data.

Supplementary Figure 3:

UMAP plots of study cohort using the 10,000 most variably methylated loci for **A)** WGBS data (n=69) , **B)** Array data (n=69) and **C)** Combined data (n=138)

Supplementary Figure 4:

A) Bar plot depicting the CpG annotation category distribution for the 32,000 probes used for MNP development in Capper *et al.* (2018). **B)** Bar plot depicting CpG annotation category for the same probeset after removal of probes with the same CpG annotation (n=16,052). **C)** UMAP of Methylation Microarray cohort using the 5,773 most variably methylated probes that were mapped to unique genomic regions. Sample shapes depict cohort source and colours denote molecular subgroup.

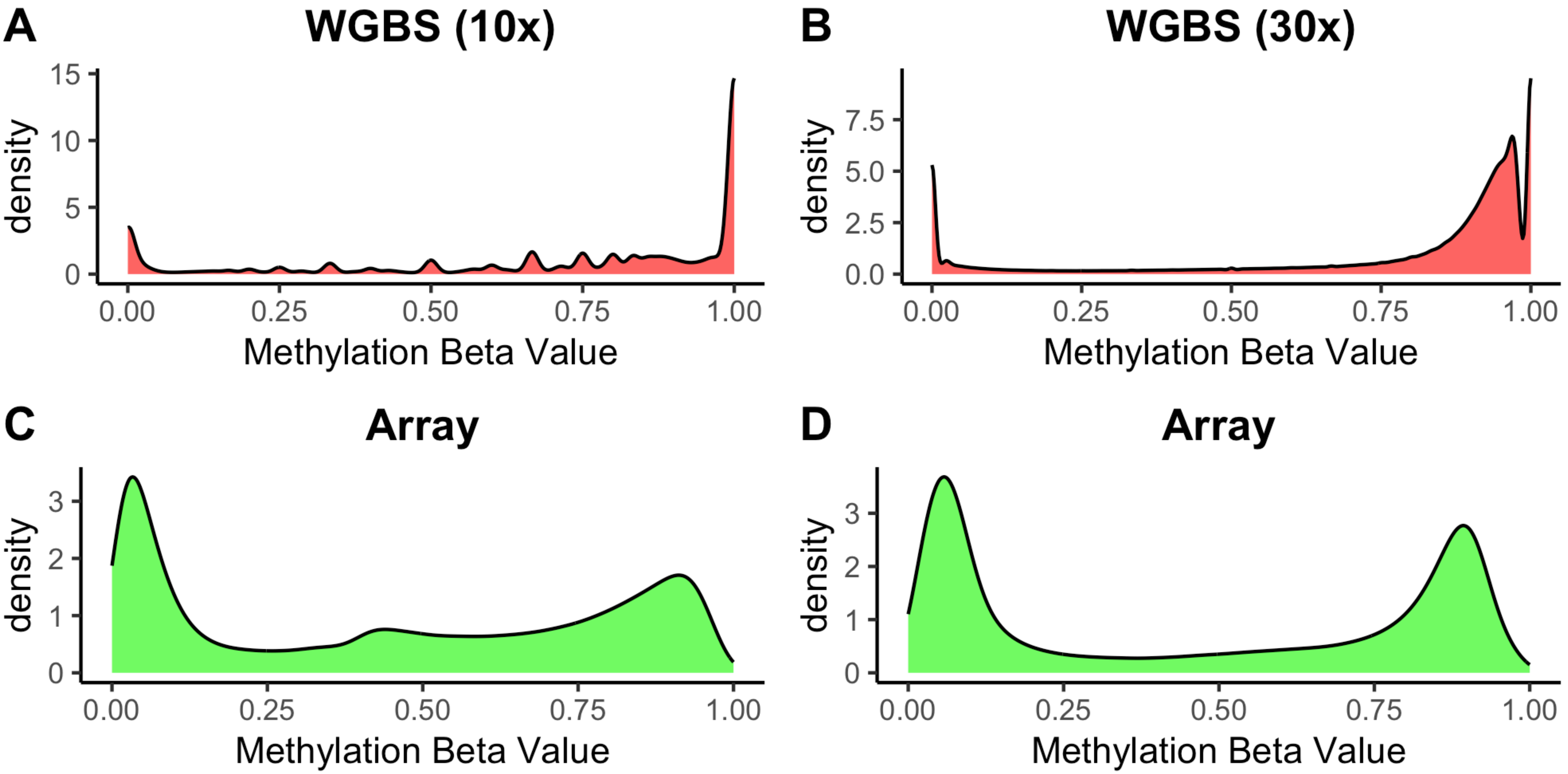

A
WGBS (10x)
B
WGBS (30x)
C
Array
D
Array
density
Methylation Beta Value
15
10
5
0
7.5
5.0
2.5
0.0
3
2
1
0.00
0.25
0.50
0.75
1.00

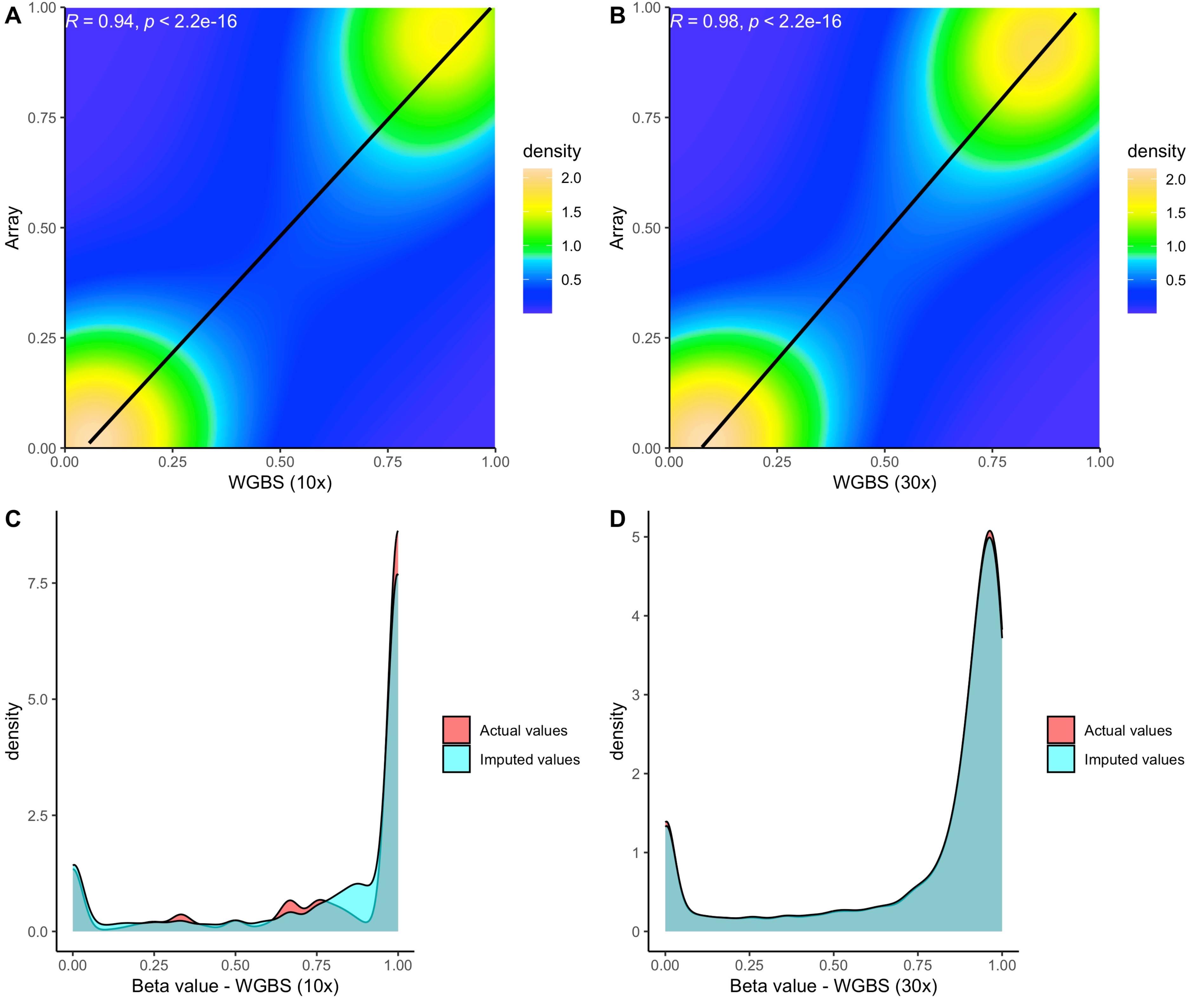

A
R = 0.94, p < 2.2e-16
Array
WGBS (10x)
density
2.0
1.5
1.0
0.5
B
R = 0.98, p < 2.2e-16
Array
WGBS (30x)
density
C
density
Beta value - WGBS (10x)
Actual values
Imputed values
D
density
Beta value - WGBS (30x)
Actual values
Imputed values

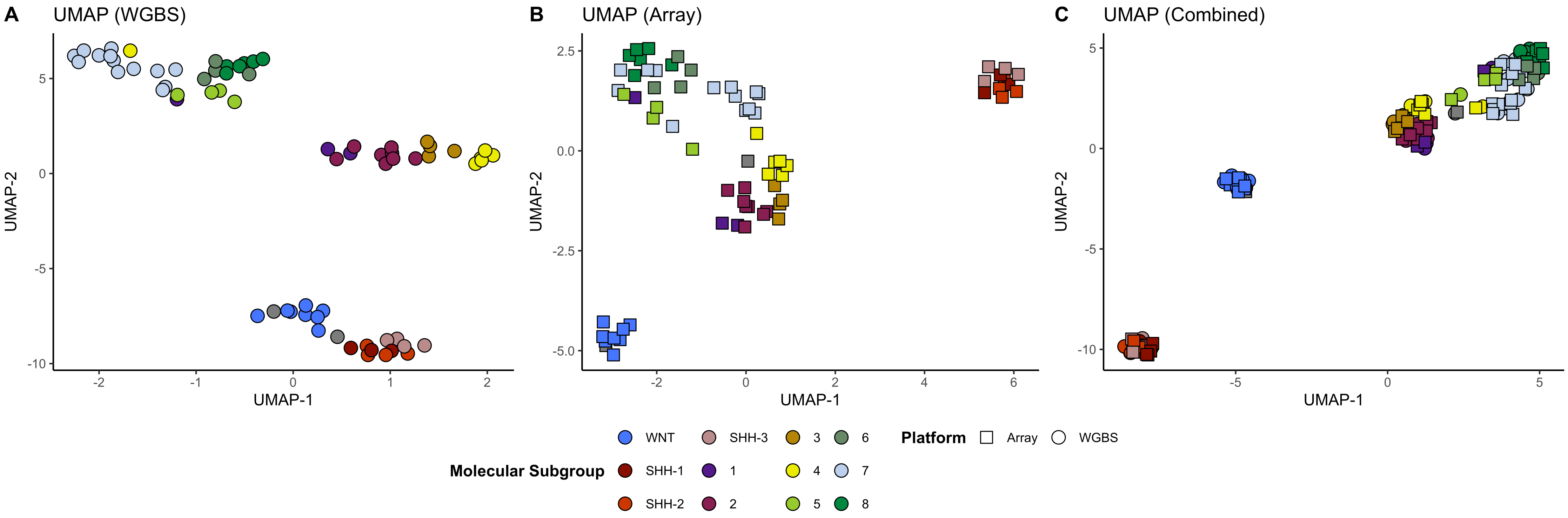
A
UMAP (WGBS)
B
UMAP (Array)
C
UMAP (Combined)
UMAP-1
UMAP-2
Molecular Subgroup
WNT
SHH-1
SHH-2
SHH-3
1
2
3
4
5
6
7
8
Platform
Array
WGBS

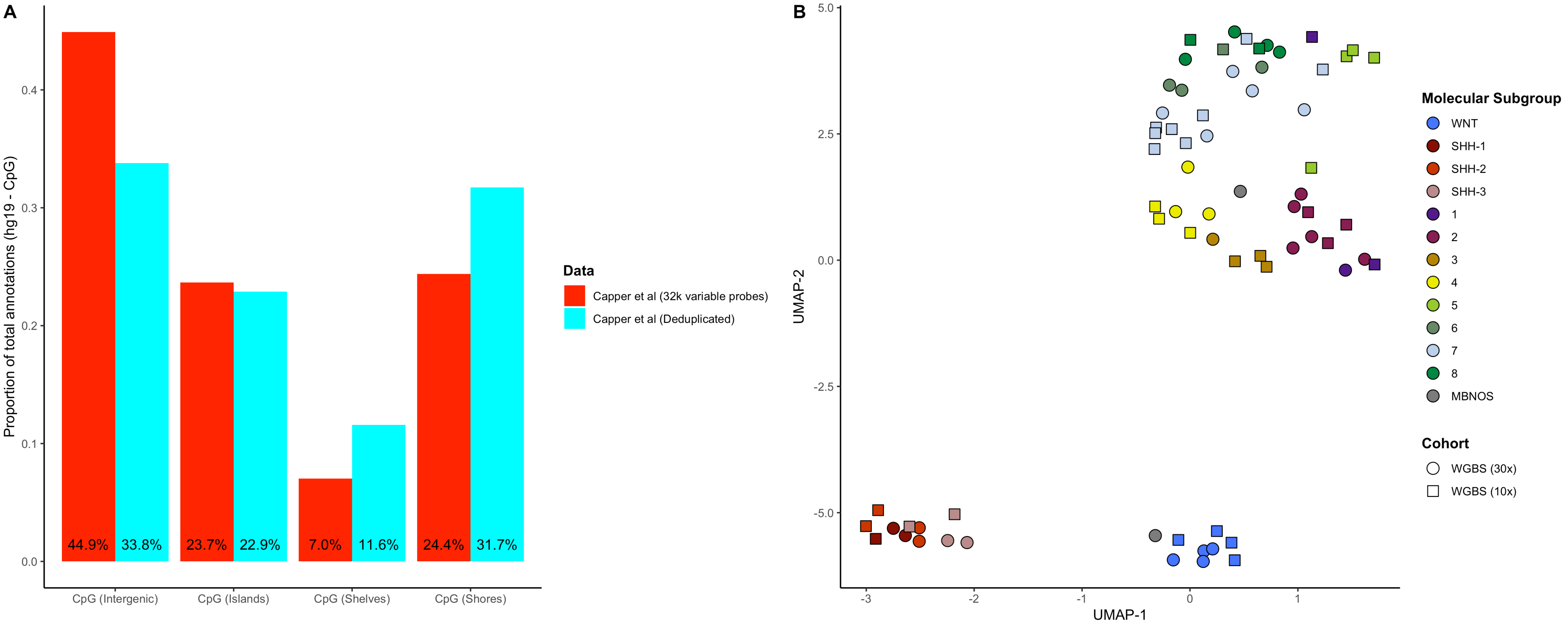
A
Proportion of total annotations (hg19 - CpG)
0.4
0.3
0.2
0.1
0.0
44.9%
33.8%
23.7%
22.9%
7.0%
11.6%
24.4%
31.7%
CpG (Intergenic)
CpG (Islands)
CpG (Shelves)
CpG (Shores)
Data
Capper et al (32k variable probes)
Capper et al (Deduplicated)
B
UMAP-2
5.0
2.5
0.0
-2.5
-5.0
-3
-2
-1
0
1
UMAP-1
Molecular Subgroup
WNT
SHH-1
SHH-2
SHH-3
1
2
3
4
5
6
7
8
MBNOS
Cohort
WGBS (30x)
WGBS (10x)
