# Supplementary material for: Robust molecular subgrouping and reference-free aneuploidy detection in medulloblastoma using low-depth whole genome bisulfite sequencing
Source: Acta Neuropathol Commun. 2025 Jun 24;13:132. doi: 10.1186/s40478-025-02049-1 (PMC12186449; doi:10.1186/s40478-025-02049-1)
Supplement: Supplementary file 2 — Supplementary material 2 [file 40478_2025_2049_MOESM2_ESM.docx]

**Supplementary Methods**

**Tumour DNA extraction**

Primary medulloblastoma tumour samples (Fresh Frozen, n=35) were prepared for sequencing. Genomic DNA (four samples = 2.5ug, 31 samples = 150ng) was extracted using the Qiagen DNeasy® blood and tissue and QIAmp® DNA FFPE tissue extraction kits respectively using the manufacturer-supplied protocols (Qiagen). All extracted DNA was eluted in DNase/RNase-free water and quantified using the NanoDrop 1000 Spectrophotometer.

**Matched array cohort summary and processing**

A cohort of matched, previously collected DNA methylation microarray samples were also used in this study for comparative analysis (n=34). For these samples, double-stranded DNA was quantified using Qubit® PicoGreen dsDNA broad-range assay kit (ThermoFisher) and analysed via IlluminaMethylation450k DNA Methylation Microarray (Bibkova et al., 2011). Beadarray IDAT files were analysed using RnBeads 2.0 using default settings (Muller et al., 2019). Probe detection p-values were checked in all samples and unreliable probes were removed using a detection p-value filter of < 0.05. Loci were filtered for potentially confounding proximal SNPs (mapping within 3 nucleotides of the assayed cytosine) and normalised via the normal-exponential out-of-band method (noob) (Fortin, Triche Jr., & Hansen, 2017). Probes located on X and Y chromosomes were removed and CpG methylation beta value matrices were prepared for downstream analysis.

**Bisulfite treatment, library preparation and sequencing**

DNA was spiked with unmethylated lambda DNA to control for unbalanced base composition and fragmented into 200-400bp lengths using the Covaris® S220 Focused-ultrasonicator. Sheared DNA fragments were then end-repaired, and bisulfite treated using the EZ DNA Methylation Gold Kit. Following bisulfite treatment, a DNA library (fragment size = 150bp) was prepared using the Accel-NGS® Methyl-Seq DNA Library Kit. Following library preparation, sample libraries were then pooled and sequenced to 10x depth using the Illumina NovaSeq 6000 via paired-end strategy.

**Sequencing data processing and quality control**

All sample data quality was summarised using FastQC version 0.11.8 (Andrews, 2010) and reads were trimmed using TrimGalore! 0.6 and Cutadapt 1.18 (Martin, 2011) (Phred score >20). Both reads 1 and 2 were trimmed by 17bp (5’ = 10bp, 3’ = 7bp) after analysing base sequence content distribution.

**Alignment, deduplication, and methylation data extraction**

Trimmed reads were aligned using Bismark version 0.22.1 (Krueger & Andrews, 2011) and Bowtie 2 (Langmead & Salzberg, 2012) to the hg19 reference genome using default settings. Reads were then deduplicated and methylation data were extracted using the ‘bismark deduplicate’ and ‘bismark methylation extractor’ functions respectively. Data were extracted in the bismark.cov format.

**External cohort summary, download and data handling.**

An additional WGBS cohort of 42, high-depth medulloblastoma (n=34) and cerebellar tissue (n=8) samples sequenced via high-depth WGBS (30x) and matched DNA Methylation Array Files (Hovestadt et al., 2014) were downloaded via the ICGC Data Portal (Zhang et al., 2019) under the project code PBCA-DE. Both WGBS sequencing and DNA Methylation Microarray data were processed as previously described, and sequencing data statistics are provided as part of the original study (Hovestadt et al., 2014). Externally acquired WGBS sample data (n=42) was downloaded in the ICGC Data Portal submission format, which was then converted to bismark.cov format, allowing both WGBS cohorts to be processed downstream in unison.

**Methylation data quality control**

Methylation read counts for both WGBS cohorts were analysed using RnBeads 2.0 (Muller et al., 2019). CpGs overlapping with known SNPs (within 3 nucleotides), high coverage outliers (defined as any CpG where coverage exceeds 50x the 0.95 quantiles of coverage values in its sample) and sex chromosome sites were removed. CpGs with fewer than 3 reads were also removed, and beta value matrices were generated for downstream analysis.
