# Supplementary material for: Robust molecular subgrouping and reference-free aneuploidy detection in medulloblastoma using low-depth whole genome bisulfite sequencing
Source: Acta Neuropathol Commun. 2025 Jun 24;13:132. doi: 10.1186/s40478-025-02049-1 (PMC12186449; doi:10.1186/s40478-025-02049-1)
Supplement: Supplementary file 3 — Supplementary material 3 [file 40478_2025_2049_MOESM3_ESM.docx]

**Supplementary Table Legends**

**Supplementary Table 1:** DNA input/quality control statistics of WGBS sequencing cohort.

**Supplementary Table 2:** CpG coverage and filtering statistics of WGBS sequencing cohort.

**Supplementary Table 3:** Molecular group and subgroup calls of primary medulloblastoma samples used in this study using the MNP classifier (n=69).

**Supplementary Table 4:** CpG annotations for the 10,000 most variably methylated CpG loci for both methylation microarray and WGBS cohorts.

**Supplementary Table 5:** CpG annotations for the 32,000 probe reference set used by Capper *et al.*
